# ‘The Wild Women of the West (Midlands)’: how LesBeWell imagined queer women’s health and its obstacles in the 1990s through the pages of *Dykenosis*

**DOI:** 10.1080/13619462.2023.2211016

**Published:** 2023-06-26

**Authors:** Hannah J. Elizabeth

**Affiliations:** Centre for History in Public Health, London School of Hygiene and Tropical Medicine and School of History Classics and Archeology, University of Edinburgh, London, UK

**Keywords:** Lesbian health, activism, National health service, mental health, sexual health

## Abstract

In 1994, the Birmingham based lesbian health activism group LesBeWell began to produce a newsletter titled *Dykenosis*. Variously describing itself as ‘for women who have sex with women’, ‘health information for dykes’ and ‘the national bi-monthly newsletter about lesbian health’, the newsletter offers a window into how one activist group imagined the health and ill health of women who had sex with women in the 1990s. By analysing *Dykenosis*, this article illuminates how LesBeWell identified and attempted to eliminate social and institutional obstacles to ‘dyke’ health. The article focuses on *Dykenosis*’ collation of experiences of invisibility and hypervisibility within Britain’s National Health Service, and the mobilisation of research, complaint, and community self-help within its pages and beyond as remedy to NHS shortcomings.

## Introduction

In October 1994 the Birmingham-based lesbian health activist group LesBeWell, printed and distributed a new lesbian health newsletter, *Dykenosis*.^1^ LesBeWell had emerged from ‘informal discussions with people working in health, [and] lesbian, gay and women’s organisations in Birmingham’, among them Lesbian Line, AIDS Lifeline, UCE Student Union and Birmingham Women’s Centre.^2^ The group's first formal meeting was in July 1994, and its newsletter *Dykenosis* soon followed.

Writing for women who had sex with women (WSW), this group of 10–12 core volunteers from a variety of backgrounds (including medicine, social care and nursing), regularly met in pubs, clubs, and homes around Birmingham to produce the provocatively named *Dykenosis*. *Dykenosis* was, obviously, an apt pun on the word diagnosis—the act of scientific discrimination or recognition of disease from its symptoms. But it was also a portmanteau of ‘dyke’ and the Greek word for knowledge or knowing. Thus it hinted at the alternative and specifically ‘dyke’ perspective on health that the newsletter would bring to its readers. LesBeWell unapologetic aim, announced through this titular pun, was that through dyke-*knowing* (information and experience), empowerment, research, and complaint, lesbians would ‘Be Well’ and the obstacles to dyke health *diagnosed*. Across 4 years and 17 pun-filled editions (which boasted 2500 readers by 1997),^3^ LesBeWell distributed ideas about health and ill-health to individual WSW, women’s groups, lesbian, gay, bisexual, trans and queer (LGBTQ) groups, and interested libraries across the United Kingdom. Over these years, *Dykenosis* described itself variously as ‘for women who have sex with women’; ‘health information for dykes’; and ‘the national bi-monthly newsletter about lesbian health’. As discussed below, following my actors, I use WSW and queer women throughout this article to acknowledge these broad definitions and to make space for the inclusion of the trans and bisexual women *Dykenosis* consciously cultivated as members of its audience. I also adopt the term ‘dyke’, as discussed later, in acknowledgement of my actors’ self-identification and the queer feminist history it evokes.

Beyond publishing *Dykenosis*, LesBeWell engaged in a number of social and campaigning activities which were integral to both the production context and much of the content of the newsletter. In its first year, LesBeWell was featured in a variety of media including national queer publications such as *The Pink Paper*, *Diva* and *Outright!*; a variety of newsletters and leaflets; union publications such as UNISON’s ‘*Out*’ and Ireland’s Union of Student’s newsletter; and the *Birmingham Evening Mail*, LesBeWell’s local mainstream newspaper.^4^ Alongside print engagement via *Dykenosis*, LesBeWell also organised and attended several Midlands and national health conferences, ran a stall at Birmingham Pride, co-ordinated a variety of health workshops, conducted two surveys into the health needs and experiences of WSW, fostered a variety of community support groups, produced a number of health leaflets, and facilitated a calendar of social and fundraising events. These events, focused on the health needs and experiences of WSW, were attended by a diverse group and included interested National Health Service (NHS) workers, academics and health civil servants, alongside *Dykenosis’* core readership of Birmingham dykes – who jovially dubbed themselves ‘The Wild Women of the West (Midlands)’.^5^

This article examines how LesBeWell represented WSW’s health and ill-health in the mid-1990s through the pages of *Dykenosis*. In particular, it focuses on LesBeWell’s collation of experiences of invisibility (presumed heterosexuality - heterosexism) and hypervisibility (homophobia and sexualisation) within Britain’s NHS and the mobilisation of research, complaint and community self-help within *Dykenosis* as a remedy to NHS shortcomings. The article begins by examining LesBeWell’s initial aims and intended audience for *Dykenosis* through the window of its first edition. It then traces how this agenda developed across the four years the newsletter ran, focusing on how it defined and attempted to ameliorate issues around mental health and sexual health. As the article shows, LesBeWell adopted a holistic approach to individual and community definitions of health throughout, discussing experiences and social conditions which led to mental and physical ill health alongside simpler informative accounts of common ailments such as cancer and thrush. As the article demonstrates, tensions between the experiential expertise of WSW regarding their own health,^6^ and medical definitions and expectations of their health, often became focal conflicts in the pages of *Dykenosis.* This was despite many of LesBeWell’s volunteers being healthcare workers and the group’s commitment to providing the latest medical facts. Throughout the analysis of *Dykenosis*, the article attends to the ways LesBeWell’s activities offer a representative but regionally specific example of WSW’s health activism in the 1990s. This opens up a new area in histories of patient activism in the period, underlining the porous nature of the NHS’ ‘universal’ service while contributing to existing histories of LGBTQ activism and patient activism.^7^ In particular the article demonstrates the multiple ways LGBTQ activist groups have attempted to identify and fulfil the health needs of specific LGBTQ communities in place of, in opposition to, and alongside, existing health and health education provided by the NHS.^8^ With LesBeWell volunteers as likely to be patients as they were healthcare providers, and with regular contributions to the newsletter offered by local NHS bureaucrats,^9^ examining *Dykenosis* demonstrates the gains to be found in paying attention to regionally specific queer activism. It also highlights the muddy divide between institutions and the activists who worked to improve them. In exploring the construction of ‘dyke health’ through the pages of *Dykenosis*, the article also offers insights into the historical and geographical contingency of these ideas and how they interacted with patients’ expectations of the NHS as an arm of the welfare state, here as mobilised and lived in 1990s Birmingham.

## ‘Dyke’ and ‘lesbian’ health activism

*Dykenosis* was, according to its subtitles, ‘for women who have sex with women’ (issues 1–5); ‘dykes’ (issues 6–14); and anyone concerned with ‘lesbian health’ (issues 15–17). Within the pages of *Dykenosis*, myriad terms proliferated as community catch-alls, most often nodding to regional identity, gender, and sexuality. Significantly *Dykenosis* did not shy away from deploying the controversial term ‘queer’ as a self-identifier. Indeed, with typical irreverence, *Dykenosis* deployed the term ‘Feel a bit queer?’ as a double entendre when it advertised a set of erotically charged safer-sex postcards—which themselves deployed the term queer among a list of lascivious words.^10^ Interested readers were later encouraged to join the Midlands-based LGBTQ health professional and student group ‘Trust Me I’m Queer!’.^11^ Following the example of my actors, as acknowledged above, I use the inclusive and expansive terms WSW and queer women throughout this article, but the more specific term ‘dyke’ used here and deployed throughout *Dykenosis*, deserves a moment of consideration.

‘Dyke’, in the pages of *Dykenosis*, did not just mean lesbian or WSW. A reclaimed slur thrown at women presumed to be lesbians, ‘dyke’ acts in *Dykenosis* as an accolade, acknowledging the hypervisibility which accompanied the lesbian health activism LesBeWell, and *Dykenosis*’ audience, were engaged in. While ‘dyke’ eschews simple definitions, or any kind of straight etymological history,^12^ in its implication of sexual disruption, swagger, visibility, and assertion, it reinforced positive attributes for women engaged in the fight for lesbian health. Thus a ‘dyke’, in *Dykenosis*, was an ‘out’ woman who read the newsletter, trusted her experiential knowledge, wrote complaints, and defined health to include positive experiences of loving and having sex with women. Put simply, dyke was a positive term in *Dykenosis*, reserved for its most visible, activist contingent: those comfortable in their sexuality, and ready to fight for a kind of WSW’s health which included an assertive celebratory visibility. Indeed, dubbing the newsletter *Dykenosis* announced to would-be readers something of the politics in which LesBeWell intended to engage. It was also a nod to those lesbian health campaigners who had come before, embracing the use of a reclaimed slur to assert a dyke definition of lesbian health just as Lesbian Liberation had in their chapter ‘In Amerika they call us Dykes’ in *Our Bodies, Our Selves*.^13^ Consequently, *Dykenosis* marked itself as *not* for readers who were offended by the term dyke or those uncomfortable being known—or indeed, those who would not see the funny side of the excess of puns which the title foreshadowed. The humour, meanwhile, signalled to readers that in reading *Dykenosis* they would become part of a fun, sexually liberated, and self-identifying political group of dykes, while acting to exclude those who through their ‘insincerity, pomposity, stupidity’ failed to get the joke or found themselves offended outsiders.^14^ As the title signalled, the humour in *Dykenosis* was persistently satirical, pun heavy, and occasionally raunchy, whether it was reclaiming lesbian stereotypes or inverting them to lampoon homophobia. As Douglas argues, jokes are ‘a victorious tilting of uncontrol against control, … the triumph of intimacy over formality, of unofficial values over official ones’.^15^ and thus, through near constant satirical and sexual puns, *Dykenosis* established itself, and its readers, as like-minded peers battling the heterosexism and homophobia which obstructed dyke health.

When LesBeWell began printing *Dykenosis* in the early 1990s, ‘lesbian health’, as opposed to more general ‘women’s health’, was seeing a surge in scientific research interest in Britain and North America. But who were these lesbians and how was their health defined? Scientific enquiries into lesbian health necessitated the drawing of boundaries around what constituted a lesbian and what ‘normal’ lesbian health might entail. However, researchers often failed to explicitly define how they were drawing boundaries between the bodies and sexual identities they were studying, with same-sex attraction, sexual behaviour, and self-identification, all deployed as ways of identifying subjects as lesbian, gay, or bisexual for research purposes.^16^

In 1999 the US National Institutes of Health published the report *Lesbian Health: Current Assessments and Directions for the Future*, which Sullivan argues recognised lesbians specifically as ‘an identifiable and measurable … biopolitical category’ for the first time.^17^ Prior to this report offering the State’s definition of lesbian health, research into lesbian health matters in the US was largely undertaken by queer community members (with funding solicited from within the queer community), occurring alongside the wider women’s health movement which emerged as a core facet of second wave feminism in the 1960s.^18^ Indeed as mentioned briefly above, *Our Bodies Our Selves* originally included a lengthy chapter written by the group Lesbian Liberation which tackled lesbian health issues and empowerment: ‘In Amerika they call us dykes’. Despite this inclusion, and though lesbian activism was fundamental to second wave feminism, demands for recognition of specific lesbian health needs elicited some conflict with heterosexual feminists.^19^ Indeed in the decades which followed its publication, the dykes’ chapter would lose its activist title, much of its anger, and eventually be subsumed into more broadly focused section on relationships.^20^ Similar friction occurred between lesbian and heterosexual feminist activists in the UK, indeed identity politics and other forms of activism against structural violence often made awkward if not incompatible bedfellows.^21^

As was the case with Amerika’s Dykes, British lesbian groups in the 1960s, through the magazines and newsletters they produced, played a pivotal role in defining (queer) women’s health and identity for their readers. These groups and their publications also influenced academic research agendas through disseminating research, community gatekeeping, and critique.^22^ As Tooth-Murphy has argued, the Minorities Research Group publication *Arena Three* helped to construct a middle class, professional ‘middlebrow’ lesbian identity predicated upon reading and understanding the latest medical literature on sexuality.^23^ The textual communities which formed around magazines like *Arena Three* represented an invaluable way into lesbian communities for academic researchers, such magazines even offering a point of contact between would—be study participants and researchers—disseminating research findings and soliciting participation.^24^ This relationship was far from universally synergistic however, with some queer women expressing persistent distrust of the psy disciplines which they saw as instrumental to heterosexist and patriarchal violence.^25^ Observing the relationship between lesbian health politics and the psy sciences in Britain across the late twentieth century, Carr and Spandler argue activists attempted to seek and define lesbian mental health and identity through assimilation into heterosexual society and the demonstration of normative sanity in the 1960s; through pride in the 1970s; and through separatism in the 1980s.^26^ In the pages of *Dykenosis* we see the legacy of these earlier definitions of lesbian health in (at times fraught) conversation with one another. The reporting of research findings and interest in participation in *Dykenosis* echoes *Arena Three*’s ‘middlebrow’ lesbians somewhat, this is unsurprising given that a number of the newsletter’s contributors were working in, or researching, health and social care. The celebration of sexuality, emphasis on coming out, and rejection of psychiatry which formed another important (though not uncontested) voice in *Dykenosis* feels more akin to the 1970s Gay Liberation Front’s stance.^27^ While the emphasis on distinct dyke and West Midlands identities echoes some of the separatist sentiment which emerged in the feminist health movements of the 1980s. Alongside these older definitions of lesbian health and health seeking behaviour, *Dykenosis* added an emphasis on health rights claims and complaints within the NHS, forming an important new facet of visible and accessible lesbian health and citizenship particular to the 1990s.

Although influenced by the outcomes of academic research, queer communities’ enquiries into queer women’s health needs in the 1980s and 1990s tended to focus on lacunas rather than build on existing research. Of particular concern was the mental health burden imposed by homophobia; concerns around queer women’s HIV risk; difficulties accessing reproductive health technologies and services; and the health impacts of queer invisibility when it came to tackling intimate partner violence and substance dependence. So while smoking, breast cancer, and alcohol use were of general concern, mirroring academic research, queer enquiries interrogated how invisibility and hypervisibility seemingly rendered queer women at greater risk. This emphasis on the situated nature of queer health, marred by homophobia and heterosexism, led queer health activists to view health holistically and value experiential expertise as much as, if not more than, the potentially prejudiced medical sciences.

More broadly, feminist and lesbian politics shaped women's health research, with queer service users and service providers, respectively, motivated to make and meet demands by wider calls to acknowledge women’s and queer rights. Research into lesbian health was still hindered by mutual distrust however, with researchers who identified as lesbians themselves encountering obstruction from within the academic establishment and reticence from their research subjects. Moves to make the mental health professions more inclusive by addressing legacies of homophobia and heterosexism in the field of psychology in the 1990s, for example, were stymied despite a flurry of interest in lesbian mental health.^28^

While WSW’s specific health needs were increasingly recognised as a subject requiring study in the 1990s, obstacles to this area of health research and delivery were myriad. The history of violence and discrimination queer service users had experienced within the NHS cast a long shadow, especially with regard to mental and sexual health provisions.^29^ Moreover, discriminatory legislation under the Conservatives—the ban on homosexual men donating blood, Section 28, the unequal age of consent—tempered queer trust in institutions like the NHS and the Health Education Authority (HEA).^30^ Even where local need and political will were present, pushback against ‘loonie left’ councils in the media and Westminster made local governments reluctant to fund LGBTQ inclusive initiatives, including health work. For example in Birmingham, the 1986 dissolution of the West Midlands County Council seemingly had a dampening effect on local equalities legislation and funding, with the newly formed Birmingham County Council keen to distance itself from its former loonie image.^31^ The fevered media reporting around queer health issues, most prominently the prurient tone of some papers’ reportage on HIV,^32^ and earlier scandals around lesbian motherhood,^33^ also created a sense of embattlement within queer communities, sowing mistrust and creating silences. Fear of discrimination led many NHS users to keep their sexuality out of healthcare interactions, even where it might be germane. Indeed, fear of ‘confirming negative stereotypes’ led to silences around mental health particularly, for fear of confirming homophobic perceptions of the queer community as somehow innately prone to mental ill-health.^34^ Queer NHS workers also faced discrimination at work if their sexuality became known. It was into this mixture of patchy research, limited funding, misinformation, silence, and fear that LesBeWell intervened through *Dykenosis*.

## ‘Part of LesBeWell’s role will be to inform the medical profession of our rights, their responsibilities and our needs’: agenda setting in Issue 1

The first *Dykenosis* issue, funded by UNISON’s West Midlands Regional Lesbian Gay Group, was printed in October 1994. The front page of Issue One proudly announced itself to be ‘for women who have sex with women’ and ‘the newsletter of LesBeWell’.^35^ Consciously acting as a ‘taster’ for the newsletter to come, it introduced the ‘issues … and topics which will be discussed and campaigned on’,^36^ laying out the core aims of LesBeWell and the intended function of *Dykenosis*. While Issue One acknowledged that LesBeWell’s ‘emphasis started with sexual health’ new members and new ideas had broadened the remit of the group to include ‘a wider range of health issues’.^37^ LesBeWell’s intentions for the newsletter went beyond filling ‘a serious gap in information provision’ for its audience of interested dykes. LesBeWell wanted ‘to get lesbian issues on the agenda … and to provide enough information to enable us to make informed decisions about our own health’.^38^ The agenda which *Dykenosis* wanted to get lesbian issues onto was that of individual dykes and the health services more broadly. In part, LesBeWell hoped to change health service provision for WSW by empowering its audience to seek health and to ameliorate community ill-health through information provision. However, as the second column on the front page of Issue One argued:

It is, of course, not enough to give advice and information to dykes if the medical practice persists in its prejudice, ignorance and insensitive handling of gays and lesbians. Part of LesBeWell’s role will be to inform the medical profession of our rights, their responsibilities and our needs.^39^

Throughout the four precarious years which LesBeWell ran *Dykenosis*, it would fulfil this commitment ‘to inform the medical professions’ through research, lobbying and complaint. The very ‘medical professions’ *Dykenosis* aimed to inform both wrote for, and subscribed to, the newsletter, offering insider knowledge on lesbian health and the issues within the NHS which challenged it. The precision of LesBeWell’s complaining activity, and the accuracy of the health knowledge *Dykenosis* imparted across its four years of publication, was partially owed to these ‘members of the health professions’ among the group’s volunteers. Some of these health professionals identified as WSW, writing about the homophobia they encountered in their roles within the NHS and demonstrating the ways the health service failed to adequately define and protect dyke health.^40^ This allowed the newsletter to discuss experiences of homophobia and heterosexism in healthcare settings from the perspective of patients *and* health professionals, a commitment *Dykenosis* made on Issue One’s front page through a discussion of homophobic attitudes amongst US nursing students. Drawing on contemporary academic research into homophobia in healthcare settings, the article deployed quotes from nursing students to illustrate the potential for discrimination perpetrated by healthcare providers – ‘[lesbians] are sick. They are not normal human beings’.^41^ But this homophobia was juxtaposed by more optimistic coverage of the Royal College of Nursing’s statement on the care of lesbian and gay patients.^42^ Changes to service provision and attitudes towards lesbians within the NHS were thus presented as necessary, but also possible, through the application of internal and external lobbying and complaint. Subsequent issues of *Dykenosis* treated this subject in far greater depth, offering insider perspectives and solutions. Forced from her job by the homophobia she encountered after coming out, a former nurse’s article provided evidence of prejudice in the British healthcare system to *Dykenosis* readers, but she also offered solutions from within. The nurse argued:

the UKCC, the governing body for nurses, midwives and health visitors produced a code of conduct … [which] states that each patient must be treated with dignity “irrespective of ethnic origin, religious beliefs, personal attributes, the nature of their health problems or any other factor”. It would appear that it is time sexual orientation was included …^43^

This call for a change to the UKCC code of conduct was later taken up by LesBeWell as a specific issue. A lobby group was formed (advised by their health professional members) which called for a ‘clause making it a disciplinary offence to be prejudiced against gays and lesbians’.^44^

Other professionals who provided content for *Dykenosis* over the years were interested in serving what they saw as a marginalised community, writing letters and articles for the newsletter to provide information and reassurance. For example, the third issue of *Dykenosis* included a lengthy letter from the chief officer of South Birmingham Community Health Council (CHC) expressing concern about homophobia in NHS care. The letter specifically solicited complaints from *Dykenosis* readers who had experienced discriminatory treatment within the NHS.^45^ This prompted letters from other Midlands CHCs requesting similar confidential complaints in later issues. Publishing letters from outside the queer community helped LesBeWell demonstrate that while it was keen to ensure *Dykenosis* documented the dangers of hypervisibility (homophobia) and invisibility (heterosexism) within the health service, it also wanted to celebrate good practice and progress, and the gains to be found in making oneself visible to the NHS on a dyke’s own terms.

Having placed homophobia and the dearth of tailored health information for dykes front and centre on page one, the remaining three pages of Issue One sketched the ‘issues’ and ‘topics’ to be discussed and ‘campaigned on’, defining dyke health and its obstacles broadly. Under a column titled ‘why is lesbian health an issue’ *Dykenosis* lamented, ‘Too many lesbians don’t tell their GPs the whole story because they are scared to come out. Too many GPs have no idea whether we should be having smears or not’; demonstrating anxiety, lesbian and bi invisibility, heterosexism, and medical ignorance as key obstacles to queer women’s health. This ambivalence around coming out, given the gaps in GPs knowledge and potential for homophobic reactions, continued across the four years of *Dykenosis*; with being ‘out’ presented as an aim, not a requirement. This hints at a more individualistic, case by case approach to being out within LesBeWell than the Gay Liberation Front’s earlier mobilisation of ‘coming out’ as a way to seek collective liberation.^46^ It certainly demonstrates a markedly less didactic approach than LesBeWell’s London contemporaries OutRage!, a group which became infamous for threatening ‘outing’ as a form of direct action.^47^ Offering a different route to change, this column was paired with a box containing what Mold identifies to be typical patient group concerns and tactics: rights within the NHS, and how to complain when they are not honoured.^48^

The box titled ‘You, Your GP, Your Rights’ encouraged complaint by communicating a simple breakdown of patient rights ‘within the Health Service’ on one side, and instructions on the ‘grievance’ procedure on the other. This emphasis on how to complain is telling. *Dykenosis*, in seeking and recounting the personal health experiences of WSW, drew on the established feminist technique of consciousness raising,^49^ but by encouraging complaint, it attempted to move WSW’s expertise from the personal, political, but rather private realm of the newsletter or lesbian disco, into the more public realm of complaints against an institution.^50^ The failure of the NHS to recognise dyke health needs then, was not only to be resolved by building communities, sharing grievances, and deploying knowledge though practices of self-care or the provision of queer community alternatives to the NHS. Rather, by raising complaints, often backed by research as well as personal testimonies, LesBeWell hoped to change the NHS itself. Thus, in the pages of *Dykenosis*, complaint offered a kind of orderly health citizenship with an activist bent, a form of activism which was readily operationalised within the institutional mechanisms of the NHS, and one which did not even require active membership in a group like LesBeWell. But as Ahmed recognises, complaining, even within the rules, is a confrontational act. It is an intervention which makes one visible, moving beyond the private realm of self-care to demand rights, disrupting institutions and exhausting complainers. It is the affective action of ‘a desire not to reproduce … an injustice’, the manifestation of ‘a desire … for a world in which those violences; injustices, do not happen’.^51^ To complain then, is to imagine a world where the injustice you have experienced will not happen again. Dyke health, as imagined by *Dykenosis*, involved complaining *because* it involved becoming visible, changing things for the better, and imagining a world where homophobia did not affect health, and dyke health was defined by dykes themselves.

Other columns in Issue One addressed breast cancer, cervical cancer, ovarian cancer, getting pregnant through artificial insemination, and alcohol misuse and dependence, demonstrating an entwined interest in both physical and mental health. *Dykenosis*’ broad definition of health falls in line with earlier moves in feminist activism which encouraged the pursuit of self-care and defined health holistically while attempting to wrest control of women’s health and its definitions from the medical establishment.^52^ The tension between this broad definition of health and commitment to community and self-help, alongside the encouragement to work (at least partially) within the system of the NHS to pursue health rights, marks a 1990s British spin on earlier American feminist ideals of self-care. That is, while *Dykenosis* offered self-care and community organising as valid additions to the welfare state’s health provisions, it also sought to invest its readers in the NHS, positioning readers as patient consumers drawing on their rights, experiences, and valid complaints to gain the care they were entitled to. That said, LesBeWell also put great effort into enabling self-help and queer community care where NHS recognition of needs was lacking or perceived to be untrustworthy. This was especially the case for mental health provisions, but also some reproductive and sexual health services, with groups formed to support self-insemination, to research ‘lesbian friendly’ fertility clinics, and to lobby the UKCC. Indeed, while changing the NHS for the better was an underlying aim of LesBeWell’s activism, later issues of *Dykenosis* made clear that where the meeting of needs by the NHS seemed unlikely, queer friendly complementary medicine, spiritual approaches to health, community organised self-care, and private healthcare services offered legitimate alternatives to the NHS. This demonstrated an imagining of dyke health which went beyond the funded limits of the welfare state and its definitions of health and healthcare. For example, queer mental health counsellors, psychotherapists and couples’ counsellors frequently advertised in *Dykenosis*, as did Oxford’s Vita Clinic, a sexual health clinic for lesbian and bisexual women. Alongside semi-regular adverts for erotic paraphernalia from queer sex shops and queer-friendly B&Bs, *Dykenosis* also advertised a lesbian reflexologist and a complementary therapy clinic and included an aromatherapy workshop in its second conference.^53^ In the round however, advertising space in *Dykenosis* was largely given over to volunteering and activism opportunities, dyke sexual health education resources, sex toys, and health related self-help groups, hinting perhaps that it was in unapologetic sexuality and community that LesBeWell really thought health could be found when reaching beyond the NHS.

As will be argued later, there was significant ambivalence in the pages of *Dykenosis* regarding the possibility that dykes might seek mental, as well as physical, health and wellbeing through their rights to health services provided by the NHS. To have one’s right to health recognised and pursued by the NHS was to be seen as a citizen. But to have one’s mental ill health recognised in the pursuit of treatment carried a burden of risk and stigma for lesbians heightened by the history of medical violence against LGBTQ individuals, including the definition of their very identity as inherently pathological.^54^ Indeed, *Dykenosis’* first foray into the discussion of dyke mental health described ‘fear’ of ‘reinforcing negative stereotyping’ as ‘valid’. This obstructive fear of ‘letting the sisterhood down’ through admitting mental ill health became a much discussed topic in later editions.^55^ This also points to a tension within *Dykenosis* around who dyke visibility, or invisibility, was for. Coming out or complaint could benefit the individual or the community when done right. Yet here we see a hint of ambivalence around both coming out, and whether individual or community health came first.

Issue One's short column titled ‘HIV and Dykes’ highlighted the absence of research into what was considered a significant WSW’s health matter in this period. ‘There has been a great deal of discussion about lesbian transmission of HIV but very little helpful research’ it explained, before noting that there had been ‘recorded cases of lesbians becoming HIV positive though unprotected sex with men or by sharing needles, where caution is obviously needed’.^56^ In a manner echoed in later issues, *Dykenosis* thus justified the urgency of its information gathering, research, and dissemination project by highlighting a lacuna in existing work, while firmly setting an inclusive tone: some lesbians (and therefore potential *Dykenosis* audience members) were HIV positive, drug users, or had sex with men. Later issues of *Dykenosis* would more consciously delineate the broad community of WSW they hoped to inform and campaign with, offering columns and letters on biphobia, trans women’s health, mental health, racism in the health services, women with children from previous heterosexual relationships, and the experiences of older WSW, to name a few. *Dykenosis* also surveyed its readership at every opportunity as part of its objective to eliminate research gaps around WSW’s health, creating quantative and qualitative data which attended to variations in identity and experience. Indeed LesBeWell’s 1995 conference report indicated that while 87% of participants identified as lesbians, 9% identified as bisexual, and around 3% as other.^57^ Some 12% of participants had sex with men, and 17.5% had children, largely from ‘relationships with men in the past’.^58^ Ages ranged from 18 to 55, roughly half of the attendees hailed from Birmingham, and 44% of respondees ‘had experienced homophobia from some part of the Health Service’.^59^ The second LesBewell 1996 conference report again offered survey data, remarking on demographic changes between the first and second conference such as fewer women identifying as bisexual, the growing number of participants between their 30s and 40s, and the wider geographic spread represented by attendees.^60^ The second report also noted that 60% of attendees ‘defined themselves as being a health worker’, 11% ‘as having a disability’, and 95.5% ‘as lesbian’.^61^ While around a third of participants lived in Birmingham, and another third hailed from Central, Southern and Eastern England, a significant number of participants made the journey from further afield, with Wales and the North West contributing significant minorities.^62^ Clearly while LesBeWell was Birmingham based and funded, its reach, and especially the reach of *Dykenosis*, went far beyond the city’s ring roads. Survey data, focus groups and the outcome of conference workshop discussions were used to directly inform which health issues LesBeWell chose to take on and again demonstrates visibility was a core tenant of dyke health, with participation in research offered as a useful way of rendering oneself visible as data.

The final page of Issue One took the form of a notice board—indeed later issues titled it thus—with small items about the newsletter, local lesbian news, and ways to support LesBeWell arranged alongside a subscription form. This final page was dominated by a large cartoon depicting the diverse LesBeWell team arm in arm. As would be typical of *Dykenosis* illustrations, the cartoon offered an image of Birmingham’s dyke community as a friendly group, mixed in age and ethnicity, their interlocked arms implying a supportive network while their open gesture welcomed newcomers and drew attention to a ‘Thank You’ list of established supporters and funders. Taken together, the page revealed the production context of *Dykenosis* as one marked by a plurality of voices, co-production, precarious funding, and by Birmingham’s specific geography and demographics.

Readers were urged to ‘Get in touch!’ in a short column asking for ‘letters, news, ideas, suggestions, advice, information and articles’.^128^ This demonstrates a commitment to a co-production model where the newsletter would be for the community and by the community, valuing experiential expertise, as was the tradition with earlier lesbian and feminist newsletters and magazines.^63^ A later issue would make this model of production and activism more explicit, Issue Three’s Notice Board instructed:

PUT PEN TO PAPER. Use your experiences to help others—we are collecting the personal experiences of women for publication in future issues of *Dykenosis* and for campaigning purposes.^64^

The short column then went on to solicit responses from bisexuals particularly.

Community building in this manner fulfilled health and activist aims by simultaneously combating loneliness while collecting evidence and information. As later issues of *Dykenosis* would make explicit, this consciously constructed community offered more than activist comradery to the newsletter’s readers; at times it formed an alternative to the NHS itself. Soliciting readers own writing also bears similarity to popular women’s magazines where readers’ letters and communications with Agony Aunts helped build a sense of community, ensuring loyal readers and so ongoing subscribers.^65^ Indeed, (as discussed below) later issues of *Dykenosis* included a Lemon Aid^66^ problem column, answering queries by pooling LesBeWell volunteers’ knowledge in place of a named agony aunt, or asking readers for their expertise. Thus *Dykenosis* echoed women’s magazines in style, but often placed readers’ expertise on an equal footing to LesBeWell volunteers, acknowledging the community’s professional and experiential knowledge while emphasising collective problem solving.

Among the list receiving appreciation in the ‘Thank You’ column in Issue One, were Peacocks the bar, specific individuals, and ‘everyone else who has donated money, given us information, come to meetings and bought raffle tickets’, indicating to readers that participation and activism could take a variety of forms.^129^ Later issues would thank The Teddy Bear Shop, Jo Joes Bar and the Electric Cinema for donations, indicating the primacy of central Birmingham for LesBeWell’s fundraising activities. Indeed, despite the group being based around three miles from Birmingham's city centre, in the suburb Moseley, adjacent to Kings Heath (an area which was important to Birmingham’s lesbian night life in the 1990s), few social or funding events took place outside the city centre. Though work assembling *Dykenosis* took place at volunteers’ homes, as the group had no premises, LesBeWell meetings took place at Peakcocks, a bar located in Birmingham’s largest shopping centre, the Bull Ring.^67^ Likewise, reoccurring fundraising discos were located either at Peacocks or Jo Joes bar, again in central Birmingham. Such central city locations offered accessibility to those coming to events from the wider West Midlands, and hints at the growing lesbian presence in the emerging queer night-time economy of Central Birmingham.^68^ However, as became clear in later editions of *Dykenosis*, the reliance on venues that served alcohol for organisational and fundraising purposes, and the limited finances of LesBeWell generally, coloured the content of the newsletter and limited its capacity to conduct research.

Another small feature on the final page of Issue One which is worth noting is ‘Another Tongue’. This section explained LesBeWell’s intention to provide ‘*funds permitting* … leaflets about smear testing and breast self-examination in a number of languages including Urdu, Bengali, Gujarati, Punjabi and Hindi’ [my emphasis].^130^

Though small, the feature demonstrates a commitment by LesBeWell to Birmingham’s South Asian populations, and like the cartoon, marked out *Dykenosis* as a newsletter which would try to cater to Birmingham’s multicultural community. While flagging a perceived gap in local health education, it also subtly indicated the ways funding had the potential to limit LesBeWell’s impact. While Issue One was paid for by the local lesbian and gay UNISON group, and North Birmingham Health Authority helped fund the first year of *Dykenosis* (stumping up £1400 to make subscriptions within the Birmingham area free), money was always scarce.^69^ In the four years LesBeWell published *Dykenosis* the group solicited funds through community donations, car boot sales, subscriptions, and allies, including businesses that benefited from queer patronage. LesBeWell also raised funds by selling ‘Wild Women’ t-shirts (a nod to their conference branding) and by organising socials with door charges and raffle tickets.^70^ These more sociable methods allowed the group to raise money while simultaneously providing social opportunities and a sense of group membership, growing LesBeWell’s profile and increasing lesbian visibility within Birmingham’s queer nightime economy. Indeed, smiling LesBeWell members in their branded t-shirts were frequently pictured in *Dykenosis*, illustrating dyke visibility and activism in action.

### ‘Legacy of prejudice’ and ‘climate of fear’: research and community as an antidote to homophobia (in NHS mental health services)

LesBeWell’s response to WSW’s mental health needs was, for the most part, to encourage the creation of groups of likeminded dykes who could support one another through difficulties. These networks were fostered by advertising and suggesting participation in existing groups from around the UK, and by cajoling readers into starting new groups (with LesBeWell support) when a need was recognised. Here LesBeWell drew on its connections with other health activist groups and organisations, acknowledging it had readers and supporters outside the West Midlands. Engaging with groups outside the West Midlands, and occasionally organisations which catered to groups beyond the queer community, allowed LesBeWell to extend its reach as a health activist group and encourage its readers’ participation (and so increased visibility) beyond the Birmingham (queer) scene while avoiding the perceived pitfalls of NHS mental healthcare.

The mental ill health among *Dykenosis*’ readership was generally thought to be caused or exacerbated by loneliness, the closet, homophobia, and sexism. Unfortunately, the latter two issues were considered endemic within the NHS’ mental health services, rendering state provisions a largely unsuitable treatment option. The situation was not considered beyond recourse however. Over the four years LesBeWell ran *Dykenosis*, while documenting dyke mental ill health and NHS failures, the newsletter also reported research which held healthcare to account, and a proliferation of self-help groups and resources which demonstrated that help and expertise was out there, even if it was largely not located within the NHS. LesBeWell also embraced queer researchers and healthcare workers, whom they trusted to investigate and treat dyke mental health with more empathy born of experiential expertise.

During LesBeWell’s first conference in June 1995, participants attempted to define the state of dyke mental health during a dedicated workshop on NHS mental health provisions. Participants discussed existing NHS mental health services, common problems affecting dykes, experiences of seeking NHS help, and the boundaries of what constituted mental health. A workshop participant reported, ‘Mental health proved to be multi-faceted and difficult to define’ before recounting the broad conclusions of those present:

The types of discrimination we can face include treating lesbianism as the cause of mental illness and a general lack of acceptance … and awareness of how straight society’s attitudes can affect us.^71^

Other specific concerns included the additional discrimination faced by Black lesbians, the homophobia experienced by dyke mental health workers, and the absence of out mental health workers on psychiatric wards leaving queer patients ‘with no positive role models’.^72^ The vastness of the topic, and the absence of adequate research was also emphasised. In many ways this workshop seems to have set the tone for the writing around mental health in *Dykenosis*.

Individual mental health issues and situations which challenged mental health were brought to the attention of LesBeWell, and so reported in *Dykenosis*, through a combination of routes. Academic interest in lesbian mental health influenced the agenda by offering the possibility of reporting on (and in some cases disputing) scientific research findings. Ongoing debates within feminist health activism also affected the issues included within *Dykenosis*, with the definitions of what constituted a health or mental health issue relevant to the community worked out on the page in readers’ letters of congratulation, complaint, and pleas for a community engagement. LesBeWell volunteers also used *Dykenosis’* conferences and newsletter to highlight issues which were of personal interest to them, as researchers, health workers, or health service users. For example, a reader wrote to say ‘I hope you will be able to do something on the menopause, it’s an important issue for all women—dykes included’.^73^ Having justified the menopause as a relevant ‘dyke’ issue, she then went on to highlight the aspects she felt deserved specific attention: ‘HRT—its benefits and the problems’, and ‘the psychological/emotional impact of the menopause which can be quite traumatic’.^74^ Having framed the menopause holistically in terms of physical and mental impacts, as well as potential treatment options, she then proposed the creation of a ‘discussion group’ for ‘dykes who find themselves entering menopause’ to break the ‘taboo’ and offer support.^75^ This letter was met with interest and eventually a group was formed via LesBeWell to discuss the menopause and other issues affecting older WSW. Definitions of health, the causes of ill health, and ways to promote dyke health were thus debated and defined in the pages of *Dykenosis*, and the groups which formed around the publication, in a manner which often prioritised experiential knowledge over medical definitions, with facts sought from extant literature, but treated with a scepticism absent in the newsletter’s more testimonial copy.

Inevitably, given the connections between mental and physical wellbeing, the catalogue of issues discussed under the banner of mental health included experiences which fit awkwardly within discreet categories, their inclusion in discussions of mental health often warranted by disputes over aetiology. For instance, it was argued that issues with addiction were often rooted in, and exacerbated by, mental ill health related to the closet and homophobia, and that aid resolving such problems was, in turn, frustrated by these issues. Similarly, the magnifying effects of isolation brought on by homophobia, heterosexism, and the closet were discussed in the context of grief and intimate partner violence.^76^ Here the invisibility of WSW’s relationships within health and social care systems was particularly charged with exacerbating problematic experiences, leaving WSW with no recourse.

*Dykenosis* reported on mental health from its first edition, charging homophobia within the mental health services as a cause of WSW’s failure to seek or receive adequate treatment. However, despite consistent discussion of present-day systemic (and individual acts of) violence, it was not until Issue Six that the newsletter addressed the ongoing effects of community memories of historic medical violence explicitly. According to Issue Six, queer mistrust and fear of medical services (a key obstacle to dyke health) had their roots in a past marred by the violence of conversion therapy and the pathologisation of homosexuality. Relying on research from the mental health charity MIND and following the one-day conference ‘Prejudice and Pride—exploring lesbian, gay and bisexual mental health’ the lengthy article reported that although ‘outright attempts at cures are no longer prevalent, more subtle forms of anti-lesbian and anti-gay practice in mental health services are endemic’.^77^ Drawing on MIND’s ‘Breaking the Link between Homosexuality and Mental Illness’ report, the article explained poor and inappropriate care was a result of retrogressive or absent training around queer health needs:

people who seek help for mental distress encounter inadequate or discriminatory care from mental health professionals who have not been trained to revise prejudices and bad practices. … All too often gay people find that mental health professionals assume any mental health problem has something to do with their being gay when it may well be completely unrelated.^78^

The article also identified fear of homophobia, and stigma around mental ill health, within queer communities, as an obstacle to accessing care.

it is often not possible for people to talk or behave openly as lesbians or gay men in hospitals, day centres etc. Other people so mistrust mental health services that they do not seek help at all. Some find it hard to discuss mental health problems in lesbian and gay communities, where they wish to ditch the old stereotype that all gay people are desperate and suicidal can mean it is taboo to talk about distress at all.^79^

In a similar vein to the discussion at LesBeWell’s earlier conference workshop, additional discrimination experienced by some lesbians was identified, although the article added some nuance:

The system does not always understand or support … the process of making choices in a complex cultural context and often works under the assumption that homosexuality is a western phenomenon and that minority lesbians and gay men thus come from communities which are more homophobic than other groups.^80^

Similarly, the article identified misapprehensions which shaped bisexuals’ experiences of mental health within the queer community and mental health services:

far from the usual assumption that they can find life easier because they can ‘pass’ for heterosexual if need be, [they] are often assumed to be sitting on the fence and thus need counselling to make a decision one way or the other rather than accepting bisexuality as a stable identity.^81^

The final damning point reported by the article was that when queer patients entered the system, their partners could be ‘side-lined’:

MIND has received reports of mental health staff discouraging frequent visits by gay partners and being given less involvement than would be given to straight partners.^82^

In spite of the litany of failures reported, the article ends on a positive and proactive note:

There are examples of good practice being developed and people should not be put off seeking support or counselling for fear of a bad reception. Contact MIND or LesBeWell for ideas of where to go for support and look out for the forthcoming LesBeWell directory of health services!^83^

The aforementioned 'Dyke Directory’ was a £1 LesBeWell publication, it contained ‘over 100 entries covering counsellors, mail order, social groups, decorators … ’ and was advertised as ‘the essential guide for dykes in Central England’.^84^ The Dyke Directory was updated frequently with additional items recommended to LesBeWell by volunteers and *Dykenosis* readers. Alongside the Dyke Directory, LesBeWell also produced a mental health leaflet and signposted more specific mental health resources produced by other groups. For example, in response to the ‘Legacy of Prejudice’ article, Issue Seven of *Dykenosis* carried a reader’s book recommendation: *Changing our Minds,* which enumerated ‘the risks lesbians take when they enter counselling or therapy and gives reasons why therapy should not be engaged in’ .^85^ Alongside this reading suggestion, LesBeWell advertised its own ‘information leaflet detailing groups, articles and other information services relating to lesbian mental health issues’ and the (previously East Midlands based) Lesbian Information Service resource booklet ‘Lesbians, Mental Health and Therapy Resource List’.^86^ By offering multiple sources of information on dyke mental health LesBeWell was able to create a discursive space within *Dykenosis*, this dismantled taboos by allowing voices for and against NHS mental health services to coexist, filling silences around past violence and present homophobia.

There were readers who, despite LesBeWell’s constant caveats about positive experiences being possible, felt *Dykenosis’* representation of NHS mental health services was too negative. Issue Eight of *Dykenosis* featured a letter from Birmingham Women’s Counselling which challenged what they felt was the thesis in *Changing our Minds* ‘that therapy is an alternative either to finding support through friendship or to taking political action’.^87^ Instead they argued this was a ‘false choice: a therapeutic relationship can enable profound changes for lesbians struggling with distress, and may release potential both to form mutually supportive friendships and to have an impact on society’.^88^ Though Birmingham Women’s Counselling admitted that ‘therapy carries risks’ and that ‘caution’ should be exercised choosing a therapist, they also ended their letter by calling for a ‘wider debate about lesbians and therapy’.^89^ Despite interventions like this, and the many small ads carried by *Dykenosis* for specialist queer self-help, therapy and counselling, the overall tone of the newsletter when it came to mainstream NHS mental health services remained one of deep suspicion. While mental health workers who read and contributed to *Dykenosis* tried to remedy the situation, LesBeWell’s own research and the studies conducted by other activist groups and charities such as MIND demonstrated the discrimination feared by the queer community was a widespread reality. Reporting MIND’s ‘Without Prejudice’ study which ‘highlighted abuse and discrimination suffered by users of services in psychiatric hospitals and the community’, *Dykenosis* listed the following damning findings in an article titled ‘Charity pinpoints “climate of fear”’.

73% of those questioned had experienced prejudice or discrimination connected with their sexual orientation.

22% of respondents said they had experienced physical and/or sexual violence while 51% said their sexual orientation had been inappropriately used by staff to explain the causes of mental distress.

Half had been told they might have fewer mental health problems if they hid or changed their sexual orientation.^90^

The article ended by stating that negative queer mental health services experiences demonstrated both a failure in health service training and equity of care, drawing on ideas of the NHS as a universal service, and healthcare as a citizen right.

All workers in mental health services should be trained to deliver quality services to everyone, not just some members of our community.^91^

Despite the pessimism such consistently dire research findings might induce, LesBeWell remained committed to proactively advising dykes on how to guard and improve their mental health. While complaint about poor NHS care remained a constant option encouraged by LesBeWell—with information on how to complain updated as the bureaucratic regimes of the NHS evolved—self-help and community care was positioned as an alternative to the failures of mainstream mental health services.

Tailored groups and reading suggestions were offered for specific mental health problems commonly experienced by dykes, allowing *Dykenosis* to cover a broad range of topics across its 17 issues. Amongst the reoccurring topics which particularly highlighted mental health in *Dykenosis* (and LesBeWell's occasional workshops), were the experiences of: mothers, trans lesbians, Black lesbians, the elderly, women experiencing domestic abuse, drug users, the lonely, the bereaved, those suffering from eating disorders, and problem drinkers. It is on the representation of this latter issue which this article now focuses. Problem drinking offers a representative example of LesBeWell’s engagement with an individual mental health difficulty: it demonstrating how *Dykenosis* informed readers of specific risks to the queer community, attempted to offer ways to bridge gaps in NHS care, highlighted the importance of research, and campaigned for improvements.

### ‘Dykes & drinking’

Briefly covered as a concerning, common, but taboo subject in the first issue of *Dykenosis*, problem drinking among dykes was frequently revisited. Articles, research reports, testimonials, surveys, leaflets, self-help groups and conference workshops were all deployed by LesBeWell to combat problem drinking which they identified as a major health risk to the dyke community. Issue One’s brief coverage admitted ‘most of the “safe spaces” we use are pubs and clubs and alcohol is central to the social lives of many of us’ before going on to warn ‘it is remarkably easy to get into a pattern of drinking destructively’, and urged those affected to seek help.^92^ While apprehension about alcohol’s dominant presence on the queer ‘scene’ continued to play a part in discussions of problem drinking (often in conjunction with self-deprecating humour which acknowledged LesBeWell’s own reliance on pubs, bars and clubs for meetings), later *Dykenosis* discussions explored the causes and consequences of ‘drinking destructively’.

Issue Three of *Dykenosis* devoted over a page to the article ‘Dykes & Drinking’ based on MSc research by Pam Bloor, exploring the extent, causes and consequences of problem drinking among dykes. Writing as an insider, but also an expert researcher, Bloor explained both the survey her research was based upon, and the results, using a sympathetic and chatty tone which positioned her experiential expertise (as a member of the queer community) alongside her academic expertise. Of the over 120 lesbians surveyed, Bloor reported 49% regularly drank ‘more than 14 units a week—the recommended sensible limit for women’ and ‘1 in 3 … were drinking 22 units or more—a level known to be linked to increased risk to health and wellbeing’.^93^ Bloor compared this figure to the 11% of women drinking more than 14 units in the ‘general population’, admitting that while perhaps ‘lesbians are just more honest about their drinking … it does seem that many of us are regularly drinking enough to increase substantially risks to our health’.^94^ Bloor’s research indicated lesbians were aware of the issue, many of those surveyed believing lesbians drank more than straight women, with some indicating that they ‘did not see drinking or our reliance on alcohol as a completely free choice’.^95^ Two core reasons for excessive drinking were given by those surveyed:

- we have nowhere to meet people other than pubs and clubs (and precious few of those) and
- alcohol is a way of dealing with stress—some of which is the result of being dykes in a largely homophobic culture.^96^

Bloor, in line with the aetiology given for many health issues among WSW covered elsewhere in *Dykenosis*, appears to have agreed with the latter hypothesis. She reported

almost all the women in the survey believed being a dyke in today’s society is still inherently stressful. Two thirds had suffered abuse, harassment and/or discrimination as a direct result of being a lesbian. Half of us aren’t out to most people in our families, two thirds aren’t out to most people we work or go to school/college/university with. … Continually having to make decisions whether to come out or not and to whom, avoiding awkward questions and ‘managing’ conversations without letting slip or feeling able to say your lover is a woman, all takes its toll on our mental wellbeing and creates a form of stress we’ve learned to live with and even take for granted.^97^

Indeed, Bloor’s research showed a causative relationship between experiences of harassment and discrimination and increased drinking among lesbians. Bloor concluded her article by reflecting on her own experiences of drinking and cutting down, acknowledging the importance of alcohol to queer socialising, in line with her survey participants, but also the benefits of reduced intake

I drank pretty heavily … It was part of the scene – I didn’t really think about what I was doing or the effects on my body. Cutting down has helped me lose weight, get rid of a stomach ulcer and I’ve now got loads more energy.^98^

Offering something of her own health history to readers and speaking as a fellow dyke softened the didactic health message and the dire warnings offered in her article. The tone seemed to resonate with *Dykenosis’* audience, and readers eventually reached out to LesBeWell for support in setting up an alcohol self-help group. This was also a response to frequent calls in *Dykenosis* for those interested in creating regional lesbian health initiatives to seek support (financial and experiential) from LesBeWell.

Unlike the existing queer alcohol support groups frequently advertised in *Dykenosis*, the new initiative, announced in Issue 11, would be located in Birmingham (rather than London), and offer help to a ‘mixed lesbian and gay group’.^99^ The ‘self-help group’ was specifically ‘intended for those who have recognised they have a drinking problem rather than acknowledged alcoholics’, allowing attendees to self-define their needs and seek intervention from peers before receiving a diagnosis or perhaps requiring more specific medical support.^100^ Existing alcohol support groups were advertised in earlier issues of *Dykenosis*. As was a call for ‘volunteer counsellors’ targeting ‘lesbians … Black and Asian people and gay men’ from the Coventry and Warwickshire Alcohol Advisory Service, but the celebrated setting up of a new group proved to readers that LesBeWell meant business and listened to readers’ requests.^101^ Rather than complaining or highlighting existing service inadequacies, through frequent taboo-busting, engagement with research, sympathetic discussion, and support group adverts, LesBeWell used *Dykenosis* to advocate for self-help and peer support as the most effective solutions to dyke problem drinking, side-stepping the involvement of less trusted NHS mental health services. When it came to sexual and reproductive health, though *Dykenosis* supported and advertised queer community alternatives, the tone was a little more hopeful about the possibility of dyke use and improvement of NHS services.

### ‘At your cervix!’ – highlighting and filling gaps, making complaints, and conducting research

Generally, writing on sexual health in *Dykenosis* focused on informing readers, highlighting gaps in existing medical knowledge, complaint about poor practice, and endeavouring to involve its readers in research to improve knowledge. Through *Dykenosis*, LesBeWell consistently argued that healthcare practitioners and patients alike needed more, and better, information on the sexual healthcare needs of WSW. By delivering better sexual health knowledge through *Dykenosis*, new resources, and healthcare conferences, LesBeWell could empower women to access the healthcare they needed while potentially avoiding the pitfalls associated with the heterosexist, homophobic and/or sexualised gaze of some medical staff. That is, if WSW knew enough, they could have their needs met without necessarily coming out to NHS staff.

While many agreed with LesBeWell’s emphasis on sexual health, it is worth noting that a large proportion of readers expressed a desire to move away from this focus, telling a LesBeWell survey conducted during the first In The Flesh conference that they felt it was tantamount to ‘complying with the general tendency to define lesbians on purely sexual grounds’.^102^ Given the consistently raunchy puns, and the celebration of eroticised health promotion materials which continued to be hallmarks of *Dykenosis’* editorial style, it would appear that this substantial minority of readers went unheeded if not unheard. That said, while sexuality between WSW was celebrated, care was taken to arm readers with the knowledge to avoid sexualisation during medical encounters.

For example, in a feature on cervical smears, *Dykenosis’* placed readers comfort and agency before any drive towards greater visibility through coming out, telling readers ‘If you are not used to penetration, ask to insert the speculum yourself or ask for a small one. … You don’t have to come out’.^103^ Though coming out was emphasised as a way to be visible, the reasons why women might not wish to, especially in a sexual healthcare setting, were manifold and much discussed.

In *Dykenosis*, the NHS was portrayed as a bureaucratic service staffed by minority of beleaguered lesbians battling misapprehending doctors and nurses. This majority of the uninformed did not understand WSW’s health needs, and were prevented from delivering equity in care by heterosexist assumptions at best, or homophobic prejudices at worst. In a damning article titled ‘Lesbophobia in nursing’ a gay ex-nurse, and LesBeWell volunteer, shared her observations of the sexualised prejudice patients presumed to be lesbians were subjected to:

Nurses joked about how they were reluctant to carry out intimate procedures for fear of either a seduction attempt, or the patient actually enjoying it. … these prejudices clearly affected the way they responded to the patient in their care.

… not every lesbian is a pervert!^104^

In other issues of *Dykenosis* male GPs were particularly critiqued for their role in poor healthcare, often pictured in cartoons deriving sexual titillation from patient disclosures of queer sexual identity. For example, in a cartoon in Issue Four a patient was pictured asking her middle aged male doctor ‘as a lesbian, what should I be doing about my sexual health?’ to which he replies ‘what exactly do lesbians do in bed? Perhaps if I were able to observe … ’ while smirking ghoulishly and sweating profusely.^105^ While such cartoons were intended to make light of awkward and potentially risky medical encounters, the obstacle prejudice presented to disclosure, and consequently necessary health interventions, was real. The intimate nature of sexual healthcare rendered the potential for identity disclosure both likely—the sex of one’s partner is perhaps at its most relevant when a sexual health history is being solicited—and risky, with encounters with healthcare practitioners made all the more uncomfortable and perhaps dangerous in situations where patients might be required to be naked.

Indeed, *Dykenosis*’ readers, in anticipation of heterosexist or homophobic encounters, were told repeatedly

You don’t have to come out – you will probably be asked about what contraception you are using and if you are sexually active (i.e. having penetrative sex with a man) – some claim celibacy to prevent further awkward questions.^106^

While the risk of homophobia engendered by outing oneself in the clinical encounter was much discussed, so too was the risk of a failure to diagnose or prevent illness caused by the non-disclosure of sexuality to doctors, or the failure to recognise health risks among WSW by both doctors and patients.

Issue 11 of *Dykenosis* featured several pages devoted to sexual health needs under the inviting title ‘Let’s Talk About Sex’.^107^ One article, ‘Ignorance is not bliss!’, offered statistics on the prevalence of Sexually Transmitted Diseases (STDs) among WSW while lamenting the absence of adequate research about transmission, treatment, or prevention. Another, ‘New Labia, New Danger?’, emphasised ‘it’s not who you are … it’s what you do that matters… we need to be aware … especially in light of our invisibility amongst health professionals’.^108^ The article then listed a panoply of sexual practices and ways of mitigating their various STD transmission risks. Among jolly cartoons and a fleet of puns, there was also a feature titled ‘STDs: lesbian favourites’ which listed trichomoniasis vaginalis, bacterial vaginosis, and thrush, as ‘favourites’ because they were common among lesbians according to the statistics featured in the ‘Ignorance is not bliss’ column.^109^ These pieces alone provided a wealth of detailed information on the sexual health risks encountered by WSW, encouraging readers to seek medical advice, while emphasising a plurality of sexual practices as acceptable and destigmatising STDs. *Dykenosis* did not care what you did in bed, or who you did it with, but encouraged regular health checks and safer-sex where possible.

Throughout *Dykenosis*, disclosure of one’s sexual practice history, rather than sexual identity, was stressed as the ideal for interactions with partners and doctors alike. The necessity of speaking frankly to doctors was most emphasised in a full page article ‘Lesbian health needs: a professional’s view’, written by a health advisor from Birmingham’s city centre GUM clinic.^110^ Offering reassurance, and demonstrating that LesBeWell’s education efforts were not in vain, she explained: ‘there is a good level of awareness of lesbian health needs at the [Whittall Street] clinic. A colleague and I attended [LesBeWell’s health conference] *Dykenosis* in the Flesh II’. Julie André then tentatively described why coming out ensured a better health outcome:

If a woman visits the clinic but doesn’t disclose that she is a lesbian, she would still benefit in that we could give her suitable treatment for, say, thrush. But if she did come out to us, the advice we could give about preventing the infection recurring would be tailored to her own particular sexual practice.^111^

While the specificities of WSW’s ‘sexual practice’ which required ‘tailored’ advice remained subtextual in this article, perhaps demonstrating a persistent discomfort with the ins and outs of WSW’s sex lives, the required ‘tailored’ advice was offered with the usual frankness by *Dykenosis* overleaf under a short feature on thrush.

Thrush, *Dykenosis* explained:

can be passed from person to person on fingers or through oral sex. Latex gloves can be used if you are careful to only touch one person with each pair, but the only real safeguard is to abstain until the infection is gone.^112^

André’s also argued that if WSW wanted to have their health needs met, they needed to be visible:

There is recognition on our part that our services need to be accessible to all parts of the community. But it is up to lesbians if they want their own health clinic, to make their demands known.

That is the reality of how these things come into being. At the moment we’re simply not seeing the sheer numbers of women who are disclosing their sexuality to us.^113^

Even with this appeal to the activist tendencies of *Dykenosis’* audience, ambivalence about being visibly *other* in the sexual health setting persisted in Issue 11. The final page of the ‘Let’s talk about sex’ feature reassured ‘Don’t let worries about coming out prevent you going along [to the doctor]. It is better to be less than honest than to get no medical help at all’.^114^ Armed with enough knowledge gained from *Dykenosis* then, secrecy could perhaps be maintained to no ill effect when it came to personal sexual health. However, as *Dykenosis* made clear, information targeted at dykes was thin on the ground and porous because of the absence of adequate research. Moreover, if dykes wanted services which catered to them, they needed to be visible in ‘sheer numbers’.

LesBeWell filled gaps where it could through research, organising health conferences, and by producing resources, but frequently *Dykenosis* also used its problem page column ‘Lemon Aid’ to highlight lacunas, attempting to crowdsource information and agitate for research when its efforts to answer readers’ questions failed to result in definitive answers. These columns often called medical expertise into question by demonstrating the ways in which WSW were not considered in the production of knowledge and technologies of sexual health.

One *Dykenosis* reader featured in Issue 13’s ‘Lemon Aid’ asked about the pessary thrush treatment Canesten, writing ‘do we know what risks are associated with using it? What are the implications for oral sex? How often is it safe to use?’^115^ In response LesBeWell wrote

We didn’t know either so we spoke to the info. Dept. at Canestan (sic) who said: “There have been no specific trials to answer your questions about oral sex. There have been cases of people swallowing Canestan (sic) thinking it was an oral tablet but no reports of ill effects.

I imagine oral sex after your partner had used the pessary would be fairly unpleasant – it wouldn’t taste nice – and I would worry about the danger of spreading the infection.

There have also been no trials about long term use but we know women are using it monthly, for example, with no reports of adverse effects”.^116^

In writing to Canesten for clarification, LesBeWell modelled good knowledge seeking behaviour for its readers, offering an example of dykes speaking frankly to experts about specific sexual practices and their consequent health concerns, as was advised by André in Issue 11. However, the speculative answer offered by Canesten in response, and the multiple admissions of ‘no specific trials’ (which would definitively answer the reader’s question), also served to reinforce the idea that more research was needed, and dykes were potentially greater experts in their own health than the ‘experts’ who consistently failed to ask research questions which acknowledged non-penetrative sex occurred.

Similarly, a reader concerned about HIV wrote to Lemon Aid in Issue 12 asking ‘As male ejaculate is one of the major transporter of the virus, is female ejaculate equally “dangerous”?’^117^ Again LesBeWell admitted their own ignorance, locating its cause in the failure of medical research to take an interest in women’s sexual health:

The simple answer is that we don’t know. The last time female ejaculation was mentioned in *Dykenosis* (Issue 6), we suggested that there was some doubt in the medical world about whether it existed or not. Although we got letters from you saying, in no uncertain terms, that it did exist – as a rush of salty liquid – we still do not know of any research into exactly what it is.

Because of this lack of knowledge, the more subtle issues – e.g. its involvement in the transmission of HIV, are swathed in mystery.^118^

Rather than writing to an ‘expert’ to solicit an answer, on this occasion LesBeWell offered educated guesses and experiential knowledge before throwing the question back to *Dykenosis’* readers:

Common sense suggests that if someone is HIV positive, all their body fluids are going to contain the virus – what we can only guess at is the concentration and its ‘efficacy’ as a transmission vehicle. If anyone has any more definite answers we’d love to hear from you.^119^

This response underlined the position of dykes, and specifically *Dykenosis* readers, as experts in their own health. The dismissive attitude of the medical establishment about the existence of female ejaculation was here derided in the face of readers’ superior experiential expertise. Soliciting and acknowledging responses from readers also helped build a sense of community and demonstrated that while doctors might not listen to women, LesBeWell did.

LesBeWell did not merely rely on experiential expertise, its health professional members, and existing scientific knowledge, they also conducted and solicited their own research on sexual health as part of their broader research initiatives. Research was conducted during focus groups at the Dykenosis In The Flesh conferences in June 1995, and in June 1996, and through questionnaires. While *Dykenosis* readers were targeted specifically, with subscribers encouraged in 1996 to ‘look out for the questionnaires which will be arriving by post’, the newsletter also promised questionnaires would be ‘turning up in pubs, clubs community groups, etc’., once again emphasising the porous and inclusive nature of LesBeWell participation.^120^ In 1997 the advertised questionnaire was sent to readers asking:

what you are interested in. It also covers areas such as whether you are out to your GP, whether you use your GUM clinic, and what you think of *Dykenosis*. By finding out what you are interested in reading about, LesBeWell will be able to target more specifically to react to definite demand.^121^

LesBeWell even researched ‘the effectiveness of Dykenosis as a means of disseminating health information to lesbians and bisexual women throughout the UK’, although this was in part because it was hoped ‘research findings may be used to support applications for further funding’.^122^

### ‘A new life for Dykenosis: LesBeWell’s legacy

The final edition of *Dykenosis* announced the postponement of LesBeWell’s upcoming health conference and plans to turn back-copies of the newsletter into a resource and information book. Under the optimistic heading ‘A new life for *Dykenosis*’, the article announced this ambitious plan and argued that the newsletter had been a victim of its own success, and the success of other health activists:

*Dykenosis* has received an amazing reception from both individuals interested in their own health and from service providers (statutory and voluntary) who are interested in lesbian health issues

… But after seventeen issues, we really have covered most elements of health that we, as volunteers, are equipped to cover.^123^

Partly the field around LesBeWell had changed:

There are now far more lesbian health groups in the UK, far more workshops and training sessions being run, and lesbian health is now included as a matter of course in lesbian and gay events.

…

We could revisit some of the issues but there are now books available which do this just as well.^124^

Another reason LesBeWell argued a book was the best legacy for *Dykenosis* was that it would prolong the ‘shelf life’ of the newsletter, preventing ‘four years of experience … talking to lesbians, talking to health providers, reading your letters and running training events’ being ‘discarded when the next issue is produced’. In an echo of the original aims of *Dykenosis*, this ‘substantial document’ would contain:

- Health information for lesbians and bisexual women
- Information on services for service providers
- Some of the small bits of research we have picked up
- Details of resources available
- Campaigning information for those who want to change services [formatting in original]^125^

Archival evidence does not indicate these plans ever made it to fruition (perhaps suggesting it did not happen or that it had a very limited print run), but the fizzling out of LesBeWell and its newsletter does not reduce the impact it had in the four years it ran. As previously argued, the geographic reach of LesBeWell, *Dykenosis*, and its associated conferences went far beyond Birmingham, additionally the group and its publications had research, activism, and textual legacies. Information from *Dykenosis* and LesBeWell’s associated community groups was widely advertised by other queer activist groups, and articles from the newsletter also informed ongoing research.^126^ Indeed the textual community which LesBeWell created forged supportive ties between activists and researchers in the Midlands which far outlasted *Dykenosis’* four year run,^127^ and many community support groups LesBeWell helped to create had a life beyond the newsletter. Moreover, while LesBeWell and *Dykenosis* emerged into what felt like a vacuum, when LesBeWell called time on *Dykenosis* four years later it did so knowing other groups were working towards dyke health. Such impacts are harder to trace than citations and reprints.

## Conclusions

LesBeWell offers an example of 1990s British queer women’s health activism which sought to fill gaps in government health education provisions and NHS services through textual intervention, networking, research, and complaint. By studying *Dykenosis*, this article has illuminated perceived obstacles to queer women’s health identified by the newsletter’s audience and producers, offering a historical qualification to the idea of the NHS as a universal health service. Though sexual health was a focus of research by LesBeWell, it was mental health which dominated their research agenda and much of their efforts around empowerment. While LesBeWell was willing to engage with queer researchers’ efforts to increase knowledge around WSW’s mental health—thus achieving visibility through an insider—generally LesBeWell preferred to foster a supportive community of groups engaged in self-care and community care. Making oneself visible in non-LGBTQ mental health settings was generally presented a difficult and risky, with fears of ‘confirming negative stereotypes’, and somehow letting down the community, compounding these difficulties. Rarely was mental health discussed without acknowledging past and/or present structural and bodily violence towards LGBTQ communities perpetrated by mental health practitioners. Indeed, while LesBeWell was keen to ensure best practice going forward via lobbying and complaint, educating its audience on past transgressions also played a role in their mission to inform and empower readers. Forewarned was forearmed when it came to mental health, visibility was still seen as risky, and solidarity was fostered in anger.

While LesBeWell made it clear that there was more to dyke health than sexual health, the dearth of information on lesbian sexual health from formal sources such as the NHS or HEA ensured information around sex played a prominent role in *Dykenosis’* mission to arm WSW with health knowledge. Sex was also important to LesBeWell in general, with sex represented as a key aspect of being a dyke in *Dykenosis*. Knowledge about sexual health was framed as fundamental to ensuring good mental and physical health for individuals and the wider WSW community. Through broad sexual health discussions, *Dykenosis* emphasised the position of WSW as experts in their own health and health risks, while documenting the failure of doctors to recognise and meet dyke’s sexual health needs by failing to perceive such risks. Consequently, LesBeWell frequently documented failures around sexual health to express wider dissatisfaction with how health among WSW was defined more generally. Thus dyke sexual health, as represented in *Dykenosis*, offered a microcosm of NHS failings and the solutions to them: it was under-researched because of lesbian invisibility, but also risky because of homophobic sexualised encounters (hypervisibility). But such risks could also be successfully navigated through self-knowledge, and ameliorated through making oneself visible via research and complaint.

While coming out was presented as a way to be visible and to receive a high standard of sexual healthcare under ideal circumstances, LesBeWell was not coy about the risks it might involve. LesBeWell’s worked to make coming out in healthcare settings a more informed choice by raising awareness of poor care, gaps in existing medical knowledge, and informing readers about how to complain if they had a bad experience. While coming out and complaint was presented as a way to be counted, and so the sisterly thing to do, confidentiality in complaints was also assured. Educating dykes through the newsletter was only part of the battle around sexual health. *Dykenosis* argued that the sexual healthcare landscape needed to change to include dyke needs (as defined by dykes) on an equal footing to other citizens using the NHS. Dyke invisibility needed to be combatted by educating practitioners on WSW’s health needs, and WSW needed to make themselves visible (when it was safe) by coming out to their doctors and making themselves available for research. When it came to sexual health then, LesBeWell was willing to work within the system towards change, encouraging dykes to participate in the NHS as patients, complainers, and subjects of research, as far as was comfortable. Should all these endeavours fail, LesBeWell also supported and produced new empowering dyke-centred sexual health resources and community groups which allowed women to guard their own health without coming out to, or even consulting, a doctor. Within the realm of sexual health then, the risks and benefits of visibility and invisibility could seemingly be controlled for the benefit of the individual and the community.

Examining *Dykenosis* offers a glimpse into the experiences of health, and health activism, lived by a group of Birmingham dykes. This alone would make the newsletters a significant corpus for queer activist history, offering a regional spin on a historiography dominated by London and blurring dichotomies which often divide activists from professionals and the institutions they hoped to change. For historians of health, as a record of experiences, *Dykenosis* demonstrates how homophobia continued to obstruct the hoped-for universality of the NHS in the 1990s for both patients and health workers, while demonstrating the significant and varied additional services provided by communities as a compliment, or alternative to, the NHS.

## Notes

1. Where articles in *Dykenosis* were published without a named author I have chosen to cite them as authored by LesBeWell, rather than leave them anonymous, to acknowledge this production collective.

2. LesBeWell, ‘One Year On…’, 1.

3. LesBeWell, ‘Reach Our 2500 Readers’*Dykenosis*, October, 1997, 4.

4. LesBeWell, ‘Publicity’, 1.

5. LesBeWell had t-shirts sporting this moniker, which they wore to events such as Pride and health conferences.

6. For a recent definition of experiential expertise in healthcare settings see Castro et al., ‘Patients’ Experiential Knowledge and Expertise in Health Care’, 307–30; for a historical deployment see Crane, *Child Protection in England, 1960–2000*.

7. Gould, *Moving Politics*; Cook, ‘“Archives of Feeling”’, 51–78; and Mold, *Making the Patient- Consumer*.

8. A comparable regionally inflected response to queer health needs can be seen in the work of Edinburgh’s Scottish AIDS Monitor. See Colin Moore, ‘Communicating Prevention: The Scottish Experience of Health Education in the AIDS Epidemic, 1981–1996’ (unpublished MSc, University of Strathclyde, 2019).

9. Louise Kilbride, Chief Officer SBCHC, ‘Dear LesBeWell…’, *Dykenosis*, March, 1995, 3.

10. LesBeWell, ‘“Feel a Bit Queer?”’ *Dykenosis*, April, 1997 , 1.

11. Trust me I’m Queer, ‘Trust Me, I’m Queer!’, *Dykenosis*, July, 1998, 4.

12. Krantz, ‘Reconsidering the Etymology of Bulldike’, 217–21; and Spears, ‘On the Etymology of Dike’, 318–27.

13. Looff, ‘The “Dykes” Chapter’.

14. Douglas, *Implicit Meanings*, 148.

15. Douglas, 152.

16. Roberts, ‘Lesbian Health Research’, 17; and Sell and Petrulio, ‘Sampling for Public Health Research’, 31–47.

17. Sullivan, ‘A Crisis Emerges’, 220–34.

18. Stoller Shaw, ‘New Research Issues in Lesbian Health’, 125–37; and Kline, *Bodies of Knowledge*.

19. Kline, p. 37; Looff.

20. Looff, p. 25.

21. Payling, ‘City Limits’, 256–73.

22. Jennings, ‘“The Most Uninhibited Party’, 883–904; Hubbard and Griffiths, ‘Sexual Offence, Diagnosis, and Activism’, 940–53; Hubbard, ‘Lesbian Community and Activism’, 1–22.

23. Tooth Murphy, ‘Arena Three Magazine’, 185–99.

24. Spandler and Carr, ‘Lesbian Politics and Psy Professions’, 6.

25. Ibid.

26. Ibid.

27. Robinson, *Gay Men and the Left*., 69–77.

28. Comely et. al, ‘Lesbian Psychology in Britain’, 16–17.

29. Dickinson, *‘Curing Queers’*.

30. Stacey, ‘Promoting Normality’, 284–304.

31. Payling, ‘City Limits’; Knowles, ‘Establishment of a Gay Community in Birmingham’, 34–38.

32. Watney and Bolton, *Policing Desire*.

33. Jennings, ‘Lesbian Motherhood’, 570–94.

34. LesBeWell, ‘Alcohol’, *Dykenosis*, October, 1994, 3; and MIND and Stonewall, ‘Prejudice and Pride - Exploring Lesbian, Gay and Bisexual Mental Health’, *Dykenosis*, October, 1995,, 2.

35. LesBeWell, ‘Working for Health - The Bigger Picture’, *Dykenosis*, October, 1994,, 1.

36. LesBeWell, ‘First Taste’, *Dykenosis*, October, 1994, p. 1..

37. Ibid.

38. Ibid.

39. Ibid.

40. LesBeWell, ‘Lesbophobia in Nursing’, *Dykenosis*, January, 1995, 3.

41. Eliason et. al, ‘Lesbian Stereotypes’, 131–44.

42. LesBeWell, ‘Working for Health’ - The Bigger Picture’, *Dykenosis*, October, 1994, 1.

43. Ibid.

44. LesBeWell, ‘Happy New Year!’, *Dykenosis*, December, 1995,1.

45. Kilbride, Chief Officer SBCHC, ‘Dear LesBeWell…’ *Dykenosis*, March, 1995, 3.

46. Robinson, pp. 69–77.

47. Robinson, pp. 175–76.

48. Mold, ‘Patients’ Rights and the National Health Service’, 2030–38; Mold, ‘Complaining in the Age of Consumption’, 185–201.

49. Rogers, ‘Feminist Consciousness-Raising’, 3–6.

50. For a comparable example of women mobilising complaint while using a mixture of professional and experiential expertise see Payling, ‘Selling Shame’.

51. Ahmed, ‘Why Complain?’.

52. Kline.

53. Dawn, ‘Lesbian Reflexologist’, *Dykenosis* January, 1997, 4; Dawn, ‘What Is Reflexology?’ *Dykenosis*, July, 1998, 4; Carol; ‘Aromatherapy’, 3.

54. Drescher, ‘Out of DSM’, 565–75.

55. LesBeWell, ‘Alcohol’, Dykenosis, October, 1994, 3.

56. LesBeWell, ‘HIV and Dykes’, *Dykenosis*, October, 1994, 2.

57. LesBeWell, ‘The Questionnaire’, 7–8.

58. Ibid.

59. Ibid.

60. LesBeWell, ‘Facts & Figures’, 4.

61. Ibid.

62. Ibid.

63. Tooth Murphy, 185–86.

64. LesBeWell, ‘Put Pen To Paper’, *Dykenosis*, March, 1995, 4.

65. Loughran, ‘The Most Helpful Friends in the World’, 135.

66. Once again *Dykenosis* is playing with puns to grab attention. Lemon was by the 1990s fairly common slang for lesbian and somewhat less common slang for vagina. Interestingly the former use was more common in Australia, but lesbian activism, especially around health, was a transnational affair by the 1990s. Victor and Dalzell, *Dictionary of Slang*, 397.

67. LesBeWell, ‘The Core of LesBeWell’, 1.

68. ‘Gay Birmingham Remembered—The Gay Birmingham History Project’; Knowles, ‘Gay Community in Birmingham, 1967–97’.

69. LesBeWell, ‘Cash Cut’, *Dykenosis*, Spring, 1996, 1.

70. LesBeWell, ‘LesBeWell T-Shirt Offer!’, *Dykenosis*, September, 1996, 5.

71. ‘Lesbian Mental Health—the View of a Participant’, 3.

72. Ibid.

73. Anon Sutton Coldfield, ‘A Plea from a Reader’, Dykenosis, January, 1995, 2.

74. Ibid.

75. Ibid.

76. Hayes, ‘Facing Loss’, *Dykenosis*, August, 1995, 2; LesBeWell, ‘Lesbians & Violence’ *Dykenosis*, March, 1995, 2; LesBeWell, ‘Abuse’, *Dykenosis*, March, 1995, 4.

77. LesBeWell, ‘A Legacy of Prejudice’, *Dykenosis*, October, 1995, 2.

78. Ibid.

79. LesBeWell, ‘A Legacy of Prejudice’, *Dykenosis*, October, 1995, 2.

80. Ibid.

81. Ibid.

82. Ibid.

83. Ibid.

84. LesBeWell, ‘The Dyke Directory Now Available’, *Dykenosis,* October, 1995, 2.

85. Solihull, West Midlands, ‘In Response… Mental Health’, *Dykenosis*, December, 1995, 3; Kitzinger and Perkins, *Changing Our Minds*, xvi, 216.

86. Solihull, West Midlands.

87. Birmingham Women’s Counselling, ‘In Response… Mental Health’, *Dykenosis*, February, 1996, 2.

88. Ibid.

89. Ibid.

90. LesBeWell, 'Charity Pinpoints ”Climate of Fear”’, *Dykenosis*, October, 1997 1.

91. Ibid.

92. LesBeWell, ‘Alcohol’, Dykenosis, October, 1994, 3.

93. Pam Bloor, ‘Dykes & Drinking’, *Dykenosis*, March, 1995, 2.

94. Ibid.

95. Ibid.

96. Ibid.

97. Ibid.

98. Pam Bloor, ‘Dykes & Drinking’, *Dykenosis*, March, 1995, 3.

99. LesBeWell, ‘Alcohol Initiative Planned’, *Dykenosis*, November, 1996, 1.

100. Ibid.

101. LesBeWell, ‘Avid Advisors Sought’, *Dykenosis*, Spring, 1996, 1.

102. LesBeWell, ‘The Future’, 3.

103. LesBeWell, ‘At Your Cervix… a Lesson in Smugness’, *Dykenosis*, December, 1995, 2.

104. LesBeWell, ‘Lesbophobia in Nursing’, *Dykenosis*, January, 1995, 3.

105. Claire Dorer, ‘Dyketoon: What Should I Be Doing about My Sexual Health?’, *Dykenosis*, May, 1995, 2.

106. LesBeWell, ‘At Your Cervix a Lesson in Smugness’, *Dykenosis*, December, 1995, 2.

107. LesBeWell, ‘Let’s Talk about Sex’, *Dykenosis*, November 1996, 2–3.

108. LesBeWell, ‘New Labia, New Danger?’, *Dykenosis*, November 1996 2.

109. LesBeWell, ‘STDs: Lesbian Favourites’*Dykenosis*, November 1996, 2; LesBeWell, ‘Ignorance Is Not Bliss!’*Dykenosis*, November 1996, 4.

110. Julie André, ‘Lesbian Health Needs: A Professional’s View’, *Dykenosis*, November, 1996 , 5.

111. Ibid.

112. LesBeWell, ‘Let’s Talk about Sex’, *Dykenosis*, November 1996, 3.

113. André, ‘Lesbian Health Needs’: A Professional’s View’, *Dykenosis*, November, 1996, 5.

114. LesBeWell, ‘Let’s Talk about Sex’, *Dykenosis*, November, 1996, 3.

115. LesBeWell, ‘Lemon Aid: Canesten’, *Dykenosis*, April, 1997, 3.

116. Ibid.

117. Subscriber, Cornwall, ‘Female Ejaculation and HIV’, *Dykenosis*, January, 1997, 3.

118. Subscriber, Cornwall, ‘Female Ejaculation and HIV’, *Dykenosis*, January, 1997, 3.

119. Ibid.

120. LesBeWell, ‘LesBeWell Research’, *Dykenosis*, September, 1996, 1.

121. LesBeWell, ‘Calling All Dykes…’, *Dykenosis*, April, 1997, 1.

122. Ibid.

123. LesBeWell, ‘A New Life for Dykenosis’, *Dykenosis*, July, 1998, 1.

124. Ibid.

125. Ibid.

126. Fish, ‘Our Health, Our Say’, 437–53.

127. Meads et al, ‘Ten Years of Lesbian Health’ 251.

128. LesBeWell, ‘Get in touch!’, *Dykenosis*, October, 1994, 4.

129. LesBeWell, ‘Thank You!’, *Dykenosis*, October, 1994, 4.

130. LesBeWell, ‘Another Tongue’, *Dykenosis*, October, 1994, 4.
